# Molecular Mechanism Analysis of STIM1 Thermal Sensation

**DOI:** 10.3390/cells12222613

**Published:** 2023-11-12

**Authors:** Xiaoling Liu, Tianyuan Zheng, Yan Jiang, Lei Wang, Yuchen Zhang, Qiyu Liang, Yuejie Chen

**Affiliations:** 1 School of Life Sciences, Beijing University of Chinese Medicine, Beijing 102401, China; k13968214887@163.com (T.Z.); 201950009@bucm.edu.cn (L.W.); rain_dawn1108@163.com (Y.Z.); 20211221004@bucm.edu.cn (Q.L.); 2 School of Pharmaceutical Sciences, Tsinghua University, Beijing 100084, China; yanjiang615@126.com; 3 School of Pharmacy, Minzu University of China, Beijing 100081, China

**Keywords:** STIM1, Orai1, Orai3, thermal sensation, molecular mechanism

## Abstract

STIM1 has been identified as a new warm sensor, but the exact molecular mechanism remains unclear. In this study, a variety of mutants of STIM1, Orai1 and Orai3 were generated. The single–cell calcium imaging and confocal analysis were used to evaluate the thermal sensitivity of the resulting STIM mutants and the interaction between STIM1 and Orai mutants in response to temperature. Our results suggested that the CC1–SOAR of STIM1 was a direct activation domain of temperature, leading to subsequent STIM1 activation, and the transmembrane (TM) region and K domain but not EF–SAM were needed for this process. Furthermore, both the TM and SOAR domains exhibited similarities and differences between STIM1–mediated thermal sensation and store–operated calcium entry (SOCE), and the key sites of Orai1 showed similar roles in these two responses. Additionally, the TM23 (comprising TM2, loop2, and TM3) region of Orai1 was identified as the key domain determining the STIM1/Orai1 thermal response pattern, while the temperature reactive mode of STIM1/Orai3 seemed to result from a combined effect of Orai3. These findings provide important support for the specific molecular mechanism of STIM1–induced thermal response, as well as the interaction mechanism of STIM1 with Orai1 and Orai3 after being activated by temperature.

## 1. Introduction

Temperature sensation is a vital mechanism for detecting changes in both external and internal body temperature, enabling the body to maintain a balance and ensure normal metabolic functioning and survival. The perception of peripheral temperature is believed to be primarily accomplished through specialized primary sensory neurons in the somatosensory system that are responsible for sensing changes in skin temperature, such as dorsal root ganglion (DRG) cells [1]. Due to the expression of temperature–sensitive receptors, DRG neurons have a high degree of temperature sensitivity. At the molecular receptor level, the most extensively researched temperature receptors currently are a type of transient receptor potential ion channels (thermoTRPs) [2,3,4,5,6,7,8,9,10,11]. However, ongoing research has led to some controversy regarding certain members of this group. For instance, TRPA1 can be activated by cold at the molecular level [10,12], while when the TRPA1, TRPV1 and TRPM3 ion channels were simultaneously knocked out in mice, nociceptive heat sensation disappeared entirely, suggesting the heat sensation involvement of TRPA1 [13]. Moreover, the thermoTRPs family can be activated not only by temperature but also by compounds [11,14]. This suggests that these temperature–activated ion channels might indirectly contribute to thermosensation.

Compared to heat and cool sensation, warm sensation is less understood at both the cellular and molecular levels, making it a particularly intriguing area of study. Interestingly, it has been reported that the warm–sensitive receptors TRPV3 and TRPV4 are mainly expressed in keratinocytes rather than the commonly believed primary sensory DRG neurons [6,7]. Based on these findings, Moqrich et al. initially proposed a hypothesis that keratinocytes could also be involved in the somatosensory system’s temperature perception [15]. Unfortunately, knocking out TRPV3 and TRPV4 individually or simultaneously has only a minor effect on the mice’s thermosensation behavior [16,17]. Therefore, it remains to be determined whether keratinocytes play a role in thermosensation. In neurons, TRPM2 has been reported to affect warm sensation when knocked out, but it requires the presence of hydrogen peroxide to be activated by warmth. Knocking out TRPV1 at the organismal level can affect the activation of warm–sensitive neurons and result in defects in warm sensation [18], but Paricio–Montesinos et al. pointed out that warm–responsive TRP Channels, including TRPV1 and TRPM2, are not absolutely required for warmth perception, while loss or local pharmacological silencing of the cool–driven TRPM8 channel abolished the ability to detect warm [19]. These data suggest the necessity for identifying new receptors for warm sensation.

Recently, a novel warm sensor in keratinocytes called STIM1 has been identified [20,21]. STIM1 is a transmembrane protein located in the endoplasmic reticulum (ER). The N–terminal region of STIM1, located inside the ER lumen, mainly consists of a regular sequence and a calcium–binding domain called EF–SAM. In addition, STIM1 contains a single transmembrane segment (TM) and a C–terminal region located in the cytoplasm. The C–terminal region of STIM1 includes three coiled–coil domains (CC1–CC3), a SOAR region, a serine/proline–rich domain (PS), a microtubule interacting and trafficking domain, and an arginine–rich domain (K domain). The most well–known and extensively studied function of STIM1 is its role in forming a type of calcium channel with Orai1, called calcium release–activated calcium (CRAC) channel. When the levels of calcium ions in the ER decrease, STIM1, located at the ER, moves towards the plasmalemma (PM). There, it binds to Orai ion channels, opening the channel and allowing calcium ions from the extracellular space to flow into the cytoplasm. This triggers the SOCE response [22,23,24]. STIM1 has also been found to be activated directly by temperature, as indicated by the formation of STIM1 multimers after heating. When co–expressed with Orai1, the heated STIM1 can bind to Orai1 during cooling and open the channel. This results in thermally induced calcium influx, which is independent of the release of calcium ions from ER [20]. STIM1 knockout in keratinocytes has been shown to shift the optimal thermal preference in mice at the physiological level from approximately 32 °C to 34 °C, which can impact their temperature selection behavior. These findings provide important insights for a deeper understanding of the precise thermosensory mechanisms in mammals. However, the specific molecular mechanism of STIM1’s temperature sensing and how it couples with Orai ion channels remain unclear. Interestingly, STIM1/Orai1 has been found to mediate thermal responses during cooling (heat–off response), while STIM1/Orai3 mediates thermal responses during both heating (heat–on response) and cooling processes. Additionally, while STIM2 shares structural similarities and is highly homologous to STIM1, overexpression of STIM2 and Orai1 in cells did not elicit a thermal response [21]. This suggests that the temperature sensitivity exhibited by STIM1 is unique. This finding provides a solid foundation for investigating the precise mechanisms underlying STIM1’s thermosensation. We generated a variety of chimeric proteins by fusing STIM1 and STIM2, as well as Orai1 and Orai3, and evaluated the thermal sensitivity of the resulting STIM mutants as well as the interaction between STIM1 and Orai mutants in response to temperature. Our primary objective was to identify the key sites of STIM1 that are responsible for temperature response and to elucidate the coupling mechanism with Orais following thermal activation. This study provides a comprehensive theoretical foundation for understanding the role of STIM1 as a thermosensor in the body and will offer valuable insights for investigating other temperature receptors.

## 2. Materials and Methods

### 2.1. Reagents and cDNA Clones

To create fluorescently tagged proteins, GFP–tagged STIM1 and STIM2, Dsred–tagged Orai1 and mCherry–tagged Orai3 were inserted into a phage plasmid. To generate STIM1/STIM2 and Orai1/Orai3 chimeras, specific residues of STIM1 were substituted with the homologous sequence of STIM2, and specific residues of Orai1 were replaced with the homologous sequence of Orai3 based on homologous sequence alignment. Using the one–step cloning kit and following the instruction manual (Vazyme Biotech, Nanjing, China), we subcloned GFP–STIM1–ΔK (residues 1–671 of STIM1), GFP–STIM1–K2 (residues 1–671 of STIM1 and 817–833 of STIM2), GFP–STIM1–SOAR2 (residues 1–343 of STIM1, 436–535 of STIM2 and 443–685 of STIM1), STIM1–R426L (change the 426th amino acid from R to L in the whole length of STIM1), mCherry–Orai3–TM–Orai1 (residues 1–62 of Orai3, 88–258 of Orai1 and 268–295 of Orai3), Dsred–Orai1–TM–Orai3 (residues 1–87 of Orai1, 63–267 of Orai3 and 259–301 of Orai1), mCherry–Orai3–TM23–Orai1 (residues 1–92 of Orai3, 118–197 of Orai1 and 173–295 of Orai3), Dsred–Orai1–TM23–Orai3 (residues 1–117 of Orai1, 93–172 of Orai3 and 198–301 of Orai1), and Dsred–Orai1–loop2–Orai3 (residues 1–140 of Orai1, 116–149 of Orai3 and 175–301 of Orai1) based on the above–mentioned plasmids containing STIM1, STIM2, Orai1 and Orai3. Additionally, STIM1 (209–685)–YFP (residues 209–685 of STIM1), STIM1–CT (233–685)–YFP (residues 233–685 of STIM1), STIM1–C227W–CFP, STIM1–N234E–CFP, CC1–SOAR–YFP (residues 238–442 of STIM1), YFP–SOAR1 (residues 344–442 of STIM1), STIM1–G379E–YFP, STIM1–F394L–YFP, STIM1–F394H–YFP, Orai–R91W–CFP, Orai1–E106A–CFP, Orai1–R83E–CFP, Orai1–E149R–CFP and Orai1–R83E–E149R–CFP were generously provided by Professor Youjun Wang (Beijing Key Laboratory of Gene Resource and Molecular Development, College of Life Sciences, Beijing Normal University), and were all inserted into the pIRESneo plasmid. These plasmids provided by Professor Youjun Wang have been well characterized before for their roles in SOCE responses.

### 2.2. Cell Culture and Transient Transfection

Human Embryonic Kidney 293T (HEK293T) cells or Hela cells were generously provided by Bailong Xiao’s lab at Tsinghua University (Beijing, China), which came from the American Type Culture Collection (ATCC, Manassas, VA, USA). These cell lines were cultured in DMEM medium supplemented with 10% FBS (Vistech, Sydney, Australia) and 1% penicillin/streptomycin (Gibco, NY, USA) in a T75 flask (Corning, NY, USA) for passaging. For transfection, cells were seeded onto poly–D–lysine–coated glass coverslips with a diameter of 8 mm (HEK293T) or 25 mm (Hela), which were placed in 24–well (8 mm) or 6–well (25 mm) plates. Lipofectamine 2000 (Invitrogen, Carlsbad, CA, USA) was used for transfection according to the manufacturer’s instructions. After approximately 24 h, cells were observed and recorded. 

### 2.3. Fura–2 Single–Cell Ca^2+^ Imaging

HEK293T cells were transfected with the relevant plasmids (1 μg DNA:1 μL lipofectamine 2000) and subsequently loaded with Fura–2 AM calcium indicator dye (Invitrogen) in Ca^2+^ imaging buffer for 30 min at room temperature. The imaging buffer contained 1 × hanks balanced salt solution (HBSS, 1.3 mM Ca^2+^_o_) and 10 mM HEPES. The Fura–2 AM was dissolved in DMSO and added, along with Pluronic™ F–127 acid (Invitrogen), to the calcium imaging buffer to prepare the loading buffer. The final concentration for both Fura–2 AM and Pluronic™ F–127 acid was 2.5 μg/mL. After being loaded with Fura–2 AM, the cells on a coverslip were placed in a chamber attached to the microscope. The change in cytoplasmic Ca^2+^ concentration was recorded using single–cell calcium imaging, while the temperature of the extracellular buffer was adjusted from 25 °C to approximately 50 °C. A Nikon microscope with a 20× objective was used for the single–cell calcium imaging, and the intracellular Ca^2+^ concentration was determined by calculating the 340/380 ratio of Fura–2. The temperature of the bath solution was controlled using a CL–100 temperature controller (Warner Instruments, CA, USA) and an SC–20 Solution In–Line Heater/Cooler (Harvard Apparatus, Holyoke, MA, USA), with temperature monitoring facilitated by a thermistor at the perfusion outlet.

### 2.4. Confocal Microscopy

HeLa cells that had been transfected with GFP–STIM1 and mCherry–Orai3 were cultured on 25 mm round coverslips. These were then mounted onto a Nikon Ti–2 Fully Motorized Inverted Microscope and continuously perfused with 1.3 mM Ca^2+^ buffer (1 × Hanks Balanced Salt Solution (HBSS, Gibco, NY, USA, 1.3 mM Ca^2+^) supplemented with 10 mM HEPES (Sigma, St. Louis, MO, USA)). The co–localization of STIM1 and Orai3 was recorded while the temperature of the extracellular buffer was adjusted from 25 °C to approximately 50 °C. Temperature control of the bath solution was achieved by using a CL–100 temperature controller (Warner Instruments, CA, USA) and an SC–20 Solution In–Line Heater/Cooler (Harvard Apparatus, Holyoke, MA), with a thermistor at the perfusion outlet to facilitate monitoring. The Z–stack module was utilized to capture GFP and mCherry fluorescence images through a 100× oil dipping objective. Recordings were taken every 15 s over a total duration of 10 min. Pictures were collected during each phase, including baseline, heat–on, and heat–off (cooled to room temperature after heating). Pearson correlation was employed to calculate the co–localization of STIM1 and Orai3.

### 2.5. Data Analysis

The data presented in all figures were expressed as mean ± SEM. Statistical significance was determined using either an unpaired Student’s *t*–test to compare differences between two samples or a one–way ANOVA to compare three or more samples. Statistical significance was denoted by * for *p* < 0.05, ** for *p* < 0.01, and *** for *p* < 0.001.

## 3. Results

### 3.1. The EF–SAM Domain Responsible for ER Calcium Sensing Is Not Essential for STIM1–Mediated Thermal Response

**Figure 1:**
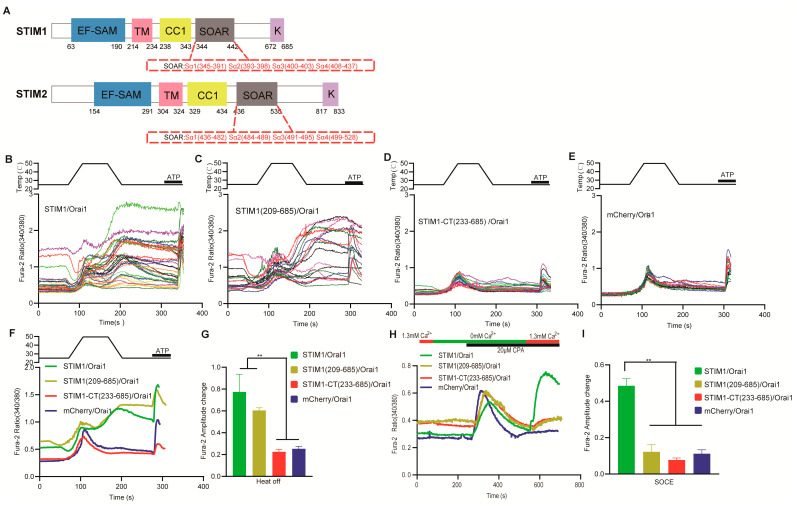
The role of STIM1 N–terminal and transmembrane (STIM1–TM) domain in temperature sensation. (**A**) Structure and sequence of STIM1 and STIM2 molecules. (**B**–**E**) Representative single–cell Ca^2+^ imaging of HEK293T cells transfected with STIM1/Orai1 (35 cells), STIM1 (209–685)/Orai1 (24 cells), STIM1–CT (233–685)/Orai1 (22 cells) and mCherry/Orai1 (17 cells). The imaging revealed the cellular response to a heating pulse, raising the temperature to approximately 50 °C, as well as the presence of ATP (500 μM) in a 1.3 mM Ca^2+^ environment. (**F**) Representative average traces and (**G**) statistical analysis of heat responses upon cooling (heat–off response) induced by the indicated constructs (STIM1/Orai1: *n* = 6 coverslips, STIM1 (209–685)/Orai1: *n* = 5 coverslips, STIM1–CT (233–685)/Orai1: *n* = 9 coverslips, mCherry/Orai1: *n* = 6, coverslips). (**H**) Representative average traces and statistical analysis (**I**) of the indicated constructs for SOCE measurement using single–cell Ca^2+^ imaging in the presence of 1.3 mM Ca^2+^ (STIM1/Orai1: *n* = 5 coverslips, STIM1 (209–685)/Orai1: *n* = 3 coverslips, STIM1–CT (233–685)/Orai1: *n* = 3 coverslips, mCherry/Orai1: *n* = 3 coverslips). The amplitude change in heat–off response in Fura–2 was determined by subtracting the initial baseline ratio (340/380) from the peak value observed during the cooling process. Similarly, the amplitude change in SOCE in Fura–2 was determined by subtracting the initial baseline ratio (340/380) from the peak value observed after changing the calcium buffer from 0 mM to 1.3 mM Ca^2+^. The baseline quantification was obtained by calculating the average value of the fura–2 ratio recorded in the first 20 s. Data are expressed as x ± s. ** p < 0.01.

STIM1 can be activated by heat and form puncta without store depletion, and STIM1/Orai1–mediated calcium influx is induced upon cooling as a heat–off response [20]. This phenomenon suggests that the N–terminal of STIM1 (Figure 1A), which contains the EF–SAM domain for ER calcium sensing, may not be a key domain for STIM1–dependent temperature sensation [25]. To test this hypothesis, we conducted experiments on the thermal response of STIM1 (209–685), which lacks the N–terminal of STIM1. Cells transfected with STIM1 (209–685)/Orai1 exhibited similar heat–off responses to those transfected with STIM1/Orai1 (Figure 1B–C,E–G), suggesting that STIM1 can be activated by temperature even in the absence of EF–SAM domain. This is a significant departure from the SOCE reaction induced by STIM1 (209–685)/Orai1 (Figure 1H,I) and clarifies why temperature–induced STIM1 clustering can occur without store depletion.

### 3.2. The Transmembrane Domain of STIM1 Was Needed for STIM1/Orai1 Temperature Response

**Figure 2:**
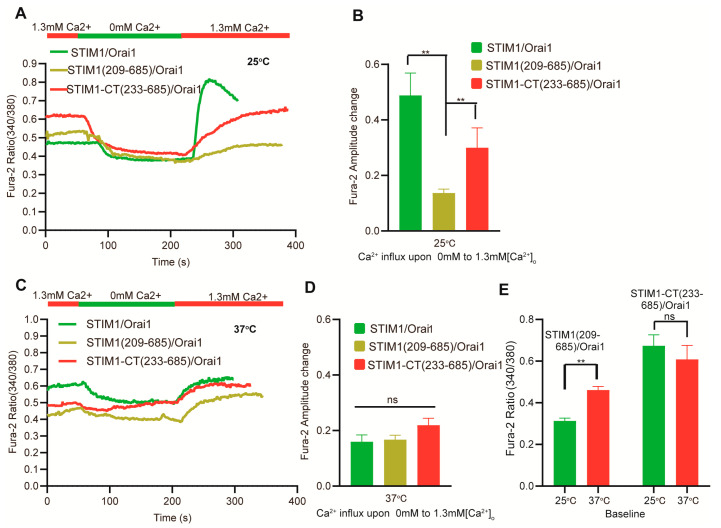
There are differences observed in basal calcium influx from 0 mM to 1.3 mM Ca^2+^ between STIM1–CT (233–685)/Orai1 and STIM1 (209–685)/Orai1. (**A**,**C**) Representative average traces and statistical analysis (**B**,**D**) of the indicated constructs for Ca^2+^ influx under the indicated bath temperatures upon switching Ca^2+^ from 0 mM to 1.3 mM (*n* = 3 coverslips for each construct). (**E**) Statistical analysis of the indicated constructs for the baseline between 25 °C and 37 °C (*n* = 3 coverslips for each construct). The fura–2 amplitude change was obtained by subtracting the baseline value in 0 mM Ca^2+^ from the peak value in subsequent 1.3 mM Ca^2+^ buffer. The baseline was obtained by calculating the average value of fura–2 ratio recorded in the first 20 s. Data are expressed as x ± s. ** p < 0.01.

Considering the unimportant role of the N–terminal in STIM–mediated temperature response, we next further truncated STIM1 to confirm the shortest segment of STIM1 that retains temperature response. Interestingly, STIM1–CT (233–685), which lacks both the EF–SAM and TM functional domains in STIM1, could not be activated by temperature and did not exhibit a heat–off response when co–transfected with Orai1 (Figure 1D–G). As was expected, STIM1–CT (233–685)/Orai1–mediated SOCE was also not observed because of the lack of EF–SAM domain for ER calcium sensing (Figure 1H,I). These findings clearly demonstrate that the TM functional domain is an essential component of STIM1 for thermal activation. We next examined the basal calcium influx of STIM1–CT (233–685)/Orai1 and STIM1 (209–685)/Orai1 from 0 mM to 1.3 mM Ca^2+^ at different temperatures (Figure 2). Although both mutations resulted in decreased calcium influx from 0 mM to 1.3 mM Ca^2+^, STIM1 (209–685)/Orai1 exhibited significantly lower calcium influx than STIM1–CT (233–685)/Orai1 at 25 °C (Figure 2A,B). Interestingly, this difference disappeared at 37 °C (Figure 2C,D), indicating that the role of the TM domain in calcium influx can be influenced by temperature. Similarly, as the temperature rose, the baseline of fura–2 ratio (340/380) in STIM1 (209–685)/Orai1 overexpressed cells was also increased, which was different from STIM1–CT (233–685)/Orai1 (Figure 2E). The TM domain appeared to have a somewhat inhibitory effect on STIM1/Orai1–triggered calcium influx at room temperature.

**Figure 3:**
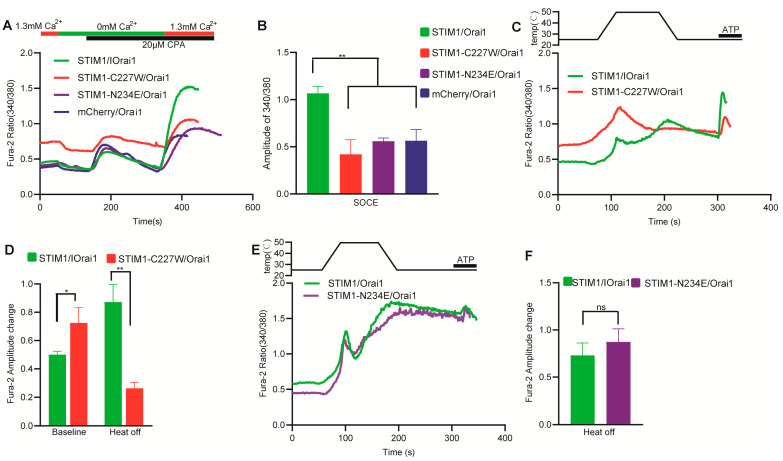
The role of single base mutations in TM domain for STIM1–induced temperature sensation. (**A**) Representative average traces of the indicated constructs for SOCE measurement using single–cell Ca^2+^ imaging in the presence of 1.3 mM Ca^2+^. (**B**) SOCE summary analysis (*n* = 3 coverslips respectively for each indicated construct). (**C**,**E**) Representative average traces by single–cell Ca^2+^ imaging of the indicated constructs in response to a heating pulse to ~50 °C in the presence of 1.3 mM Ca^2+^. (**D**) Statistical analysis of the indicated constructs for baseline and heat–off response (STIM1/Orai1: *n* = 6 coverslips; STIM1–C227W/Orai1: *n* = 5 coverslips). (**F**) Statistical analysis of the indicated constructs for heat–off response (STIM1/Orai1: *n* = 5 coverslips; STIM1–N234E/Orai1: *n* = 4 coverslips). The baseline was obtained by calculating the average value of the fura–2 ratio recorded in the first 20 s. The fura–2 amplitude change in heat–off response was obtained by subtracting initial baseline from the peak value during cooling process. The fura–2 amplitude change in SOCE was obtained by subtracting initial baseline from the peak value after changing calcium buffer from 0 mM to 1.3 mM Ca^2+^. Data are expressed as x ± s. * p < 0.05, ** p < 0.01.

To further clarify the role of the TM domain in STIM1–dependent temperature sensation, we conducted tests on two STIM1 mutants with single base mutations, namely STIM1–C227W and STIM1–N234E. The C227W mutation is known to induce constitutive Ca^2+^ influx through the CRAC channel and represents an activated state of STIM1 [26]. Our findings are consistent with this, as STIM1–C227W/Orai1 had a significantly higher baseline than STIM1/Orai1 in the beginning (Figure 3A,C,D). Cells transfected with STIM1–C227W/Orai1 did not exhibit both SOCE and heat–off response as STIM1/Orai1 (Figure 3A–D), indicating that no further SOCE response and thermal response can be activated in this open state of CRAC channel. N234 is the last amino acid of the TM domain (Figure 1A) [27] and adjoins the CC1 domain of STIM1. CC1–SOAR is an inhibitory domain for preventing STIM1 activation at rest state, and N234 is important for disinhibition of the CC1–SOAR domain in the cytoplasm during SOCE response [28]. Interestingly, STIM1–N234E/Orai1 retained the heat–off response despite having impaired SOCE (Figure 3A,B,E,F), suggesting that different signal transduction mechanisms exist for the TM domain between ER store depletion and temperature–induced STIM1 activation. This means that the disinhibition of CC1–SOAR may not depend on the TM domain for temperature activation.

### 3.3. The K Domain of STIM1 Is an Indirectly Acting Site for Temperature

**Figure 4:**
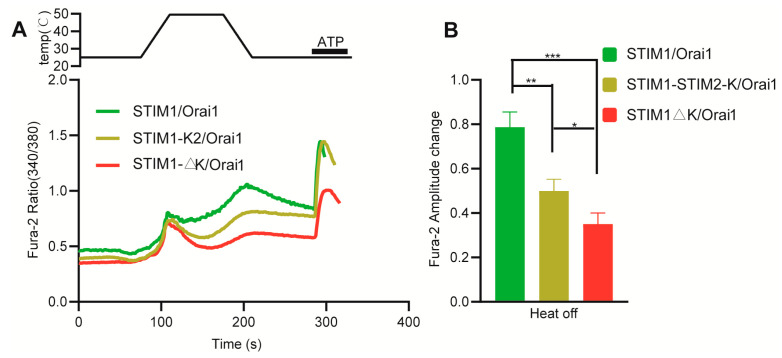
The role of K domain in STIM1 temperature sensation. (**A**) Representative average traces of HEK293T cells transfected with indicated constructs in response to a heating pulse to around 50 °C in the presence of 1.3 mM Ca^2+^ using single–cell Ca^2+^ imaging. (**B**) Statistic analysis of the heat–off responses for STIM1/Orai1 (*n* = 12 coverslips), STIM1–K2/Orai1 (*n* = 11 coverslips) and STIM1–ΔK/Orai1 (*n* = 10 coverslips). The fura–2 amplitude change in heat–off response was obtained by subtracting initial baseline from the peak value during cooling process. Data are expressed as x ± s. * p < 0.05, ** p < 0.01, *** p < 0.001.

Next, our aim was to determine the specific domain of STIM1 that directly interacts with temperature and is responsible for the initial temperature response. Our previous studies have shown that the removal of the K domain (STIM1–ΔK) led to a loss of temperature sensitivity while still preserving most of the SOCE [21]. To investigate whether temperature can directly act on the K domain of STIM1 and induce temperature response, we replaced it with that of STIM2 (STIM1–K2) to determine whether it could restore the thermal response in STIM1–ΔK/Orai1. Interestingly, STIM1–K2/Orai1 partially restored the heat–off response, but to a significantly lesser extent than that observed in STIM1/Orai1 (Figure 4). These findings imply that the K domain of STIM1 is not exclusively responsible for its thermal activation and may be considered as the indirect site of temperature activation. This is supported by the fact that STIM2, which lacks thermal sensitivity, can partially restore the thermal response of STIM1 through its K2 domain. The K domain may be employed to facilitate STIM1 anchoring from the ER to PM after temperature activation [29].

### 3.4. Temperature Acted Directly on the Inhibitory Domain of CC1–SOAR in STIM1

**Figure 5:**
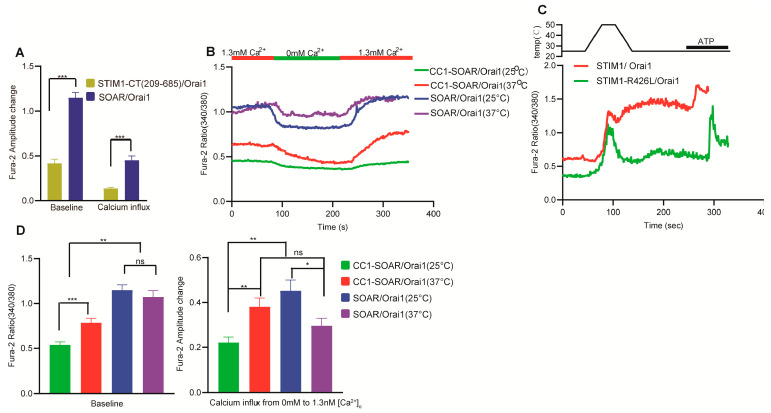
The role of temperature in CC1–SOAR inhibitory domain and Orai1 activating domain SOAR. (**A**) Statistical analysis of baseline and basal calcium influx from 0 mM Ca^2+^ to 1.3 mM Ca^2+^ at room temperature for STIM1–CT (209–685)/Orai1 and SOAR/Orai1 (*n* = 6 coverslips for each construct). (**B**) Representative fura–2 ratio of HEK293T cells transfected with the indicated constructs when changing from 0 mM Ca^2+^ to 1.3 mM Ca^2+^ at 25 °C or 37 °C. (**C**) Representative average traces of HEK293T cells transfected with indicated constructs in response to a heating pulse to around 50 °C in the presence of 1.3 mM Ca^2+^ using single–cell Ca^2+^ imaging. (**D**) Statistical analysis of baseline (left) and basal calcium influx (right) for CC1–SOAR/Orai1 (25 °C, *n* = 9 coverslips; 37 °C, *n* = 10 coverslips) and SOAR/Orai1 (25 °C, *n* = 6 coverslips; 37 °C, *n* = 6 coverslips). The baseline was obtained by calculating the average value of the fura–2 ratio in the first 20 s. The calcium influx was obtained by subtracting the baseline value in 0 mM Ca^2+^ from the peak value in subsequent 1.3 mM Ca^2+^ buffer. Data are expressed as x ± s, * p < 0.05, ** p < 0.01, *** p < 0.001.

Our results showed that changes in Ca^2+^ concentration from 0 mM to 1.3 mM Ca^2+^ led to significantly lower calcium influx in cells transfected with STIM1–CT (209–685)/Orai1 compared to SOAR/Orai1 (Figure 5A), despite the smaller size of SOAR. This is mainly due to the inhibitory functional domain of SOAR present in the CC1 region [28,30]. The CC1 region binds with SOAR in the resting state, preventing it from activating Orai1. However, STIM1 can undergo a conformational change and transmit signals from the ER to the cytoplasm through the transmembrane domain located at the ER membrane. This enables STIM1 to overcome the inhibitory state of CC1–SOAR and become activated. Once activated, the SOAR in the vicinity of the ER moves to the ER–PM junction, where it can activate Orai1 near the PM domain [31,32,33].

Based on the aforementioned study, we investigated the impact of temperature on SOAR and the inhibitory domain of CC1–SOAR. Our findings indicate that cells transfected with SOAR/Orai1 exhibited a significantly higher baseline than those transfected with CC1–SOAR/Orai1 (Figure 5B,D), suggesting an inhibitory role between CC1 and SOAR. Interestingly, we observed that SOAR/Orai1–mediated calcium influx when changing calcium concentration was smaller at 37 °C than at 25 °C, suggesting an inhibitory effect of temperature on SOAR–Orai1 interaction. This is consistent with the previous study [20]. It provides a possible explanation for the temperature response pattern of STIM1/Orai1, which is inhibited at higher temperatures and exhibits a heat–off response after cooling. Conversely, we observed a higher baseline and calcium influx when changing Ca^2+^ concentration for CC1–SOAR/Orai1 at 37 °C than at 25 °C, supporting our hypothesis that temperature can directly impact the inhibitory domain of CC1–SOAR. Specifically, higher temperatures can overcome the inhibitory effect between CC1 and SOAR, similar to the effect induced by calcium release from the ER, and promote the interaction between SOAR and Orai1. This finding was further corroborated by the results obtained from the STIM1–R426L mutation located within the CC3 domain of SOAR. We observed that STIM1–R426L/Orai1 displayed a significantly lower baseline compared to STIM1/Orai1. This aligns with a previous study indicating that the R426L mutation can enhance the inhibitory effect of CC1–SOAR, rendering STIM1 more inactive [30,34]. Interestingly, we discovered that the R426L mutation in the SOAR domain severely disrupted the heat–off response mediated by STIM1/Orai1 (Figure 5C). This could be attributed to the inhibitory effect of the R426L mutation on CC1–SOAR, making it more difficult for STIM1 to be activated by temperature. By combining this study and previous research [20,21], we conclude that temperature can directly impact the inhibitory domain of CC1–SOAR, override the inhibitory effect, and activate STIM1. However, heat can prevent the coupling of STIM1 and Orai1 via SOAR, leading to a dominant heat–off response after cooling for STIM1/Orai1.

### 3.5. Different Mechanism Exists for Interaction between SOAR and Orai1 in STIM1–Induced Thermal Response

**Figure 6:**
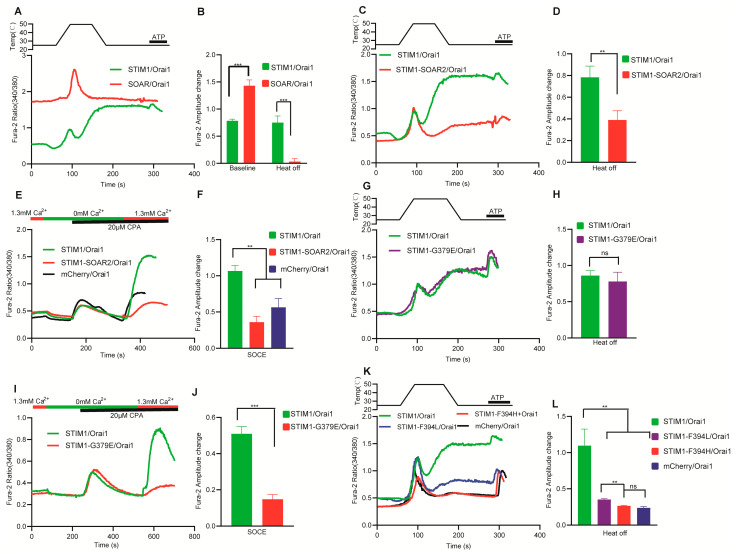
The role of SOAR domain in thermal response of STIM1/Orai1. (**A**,**C**,**G**,**K**) Representative average traces of HEK293T cells transfected with indicated constructs in response to a heating pulse to around 50 °C in the presence of 1.3 mM Ca^2+^ using single–cell Ca^2+^ imaging. (**B**) Baseline and heat off response summary (STIM1/Orai1: *n* = 9 coverslips; SOAR1/Orai1: *n* = 9 coverslips). (**D**) Statistic analysis of heat–off responses for STIM1/Orai1 and STIM1–N234E (*n* = 8 coverslips, respectively, for each indicated construct). (**E**,**I**) Representative average traces of the HEK293T cells transfected with indicated constructs for SOCE measurement using single–cell Ca^2+^ imaging. (**F**,**J**) SOCE summary analysis (**F**): *n* = 3 coverslips respectively for each indicated construct; (**J**): STIM1/Orai1 (*n* = 8) and STIM1–G379E (*n* = 3). (**H**) Statistic analysis of heat–off responses for STIM1/Orai1 (*n* = 13 coverslips) and STIM1–G379E (*n* = 4 coverslips); (**L**) Statistic analysis of heat–off responses for indicated construct, *n* = 3 coverslips for STIM1/Orai1, STIM1–F394L/Orai1, STIM1–F394H/Orai1 and mCherry/Orai1 respectively. The baseline was obtained by calculating the average value of the fura–2 ratio in the first 20 s. The fura–2 amplitude change in heat–off response was obtained by subtracting initial baseline from the peak value during cooling process. The fura–2 amplitude change in SOCE was obtained by subtracting initial baseline from the peak value after changing calcium buffer from 0 mM to 1.3 mM Ca^2+^. Data are expressed as x ± s. ** p < 0.01, *** p < 0.001.

SOAR is the minimum domain required for STIM1 activation. When co–transfected with Orai1, SOAR can elicit sustained calcium influx, as demonstrated by the significant baseline increase in SOAR/Orai1 compared to STIM1/Orai1 [35,36]. Our findings indicated that SOAR/Orai1 was not further activated by temperature (Figure 6A,B). In contrast, a higher temperature could inhibit the basal calcium influx induced by SOAR/Orai1 (Figure 5B,D), suggesting that SOAR may not be the direct site of STIM1 activation by temperature. To further investigate the function of SOAR in STIM1/Orai1–mediated thermal response, we replaced the SOAR domain of STIM1 with that from STIM2 (STIM1–SOAR2), as STIM2 could not be activated by temperature despite its high homology to STIM1. The thermal response and SOCE of STIM1–SOAR2/Orai1 were both abolished (Figure 6C–F). This may be attributed to the weaker interaction between the SOAR2 domain and Orai1 [37]. The consistent trend observed between thermal response and SOCE in the STIM1–SOAR2 chimera suggests the existence of a similar mechanism for STIM1–Orai1 interaction or activation.

We refined the mutation range of SOAR in STIM1 and examined their thermal responses in comparison to SOCE activity. Sa1, Sa2, Sa3 and Sa4 are included in SOAR (Figure 1A). Sa1 in SOAR primarily interacts with the C–terminal region of Orai1, while Sa2 is responsible for opening the Orai1 channel. Previous research has identified G379 in Sa1 and F394 in Sa2 as critical residues for stronger activation of Orai1 by STIM1 compared to STIM2 [31,37]. G379 of STIM1 is the main point for stronger interaction of SOAR with CC1 in the resting state and with Orai1 in the activated state compared to STIM2. We generated the mutant STIM1–G379E and found that the SOCE for STIM1–G379E/Orai1 was significantly reduced compared to STIM1/Orai1 (Figure 6I,J), consistent with previous studies [31]. Interestingly, we found that the heat response of STIM1–G379E/Orai1 was unaffected (Figure 6G,H), indicating that distinct interaction mechanisms existed for STIM1–Orai1 interaction between these two responses. This effect may be attributed to the increased dissociation of SOAR and CC1 triggered by temperature, and the STIM1 interaction ability with the C–terminal of Orai1 may also be enhanced. Sa2 has only one different site between STIM1 (F394) and STIM2 (L485). STIM1–F394L/Orai1 mutant was reported to have decreased SOCE, while STIM1–F394H/Orai1 lost SOCE completely. Previous studies have indicated that STIM1–F394H cannot effectively combine with Orai1, while STIM1–F394L still has the ability to combine with Orai1 [37]. Our research revealed a similar trend for thermal responses, with a significant decrease in the thermal response of STIM1–F394L/Orai1 and a complete loss of thermal response in STIM1–F394H/Orai1 (Figure 6K,L). Our findings demonstrate distinct mechanisms underlying the interaction between the C–terminal region of Orai1 and STIM1 during SOCE and thermal response. However, the process for opening the Orai1 channel appears to be similar in both pathways.

### 3.6. The Key Sites of Orai1 Played a Similar Role in STIM1–Dependent Thermal Response and SOCE

**Figure 7:**
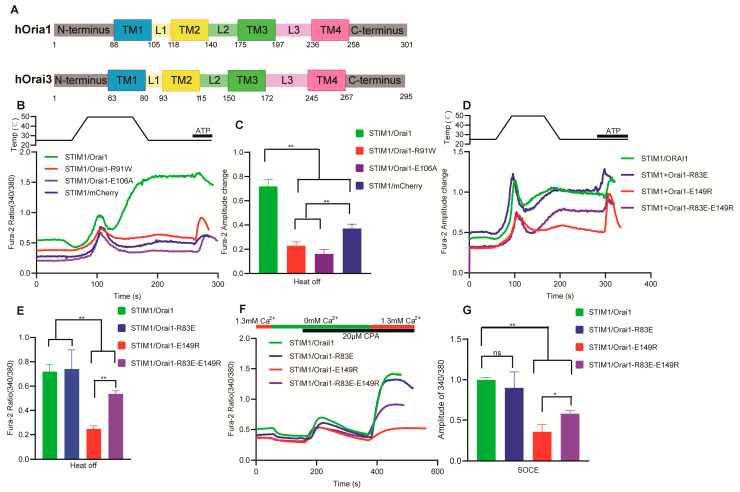
Analysis of the coupling mechanism between STIM1 and Orai1 in thermal response by key–site mutants of Orai1. (**A**) Structure and sequence of Orai1 and Orai3 molecules. (**B**,**D**) Representative average traces of HEK293T cells transfected with indicated constructs in response to a heating pulse to around 50 °C in the presence of 1.3 mM Ca^2+^ using single–cell Ca^2+^ imaging. (**C**) Statistic analysis of heat–off responses for STIM1/Orai1 (*n* = 6 coverslips), STIM1/Orai1–R91W (*n* = 3 coverslips), STIM1/Orai1–E106A (*n* = 4 coverslips) and STIM1/mcherry (*n* = 5 coverslips). (**E**) Statistic analysis of heat–off responses for STIM1/Orai1 (*n* = 6 coverslips), STIM1/Orai1–R83E (*n* = 4 coverslips), STIM1/Orai1–E149R (*n* = 5 coverslips) and STIM1/Orai1–R83E–E149R (*n* = 4 coverslips). (**F**) Representative average traces of the HEK293T cells transfected with indicated constructs for SOCE measurement using single–cell Ca^2+^ imaging. (**G**) Statistic analysis of SOCE responses for STIM1/Orai1 (*n* = 3 coverslips for each construct). The fura–2 amplitude change in heat–off response was obtained by subtracting the initial baseline from the peak value during cooling process. The fura–2 amplitude change in SOCE was obtained by subtracting initial baseline from the peak value after changing calcium buffer from 0 mM to 1.3 mM Ca^2+^. Data are expressed as x ± s. * p < 0.05, ** p < 0.01.

We next wanted to further investigate the role of Orai1 in STIM1–dependent thermal response; we focused on the representative key sites of Orai1, which are crucial in SOCE reaction, and examined the roles of these regions or sites in STIM1–dependent thermal response. The mutations Orai1–R91W and Orai1–E106A can lead to the inactivation of the Orai1 ion channel and its inability to induce calcium release–mediated calcium ion influx [38,39]. Therefore, we separately analyzed the thermal responses of STIM1/Orai1–R91W and STIM1/Orai1–E106A. Consistent with the SOCE reaction, the STIM1–related thermal response of these two mutations was completely abolished (Figure 7B,C), indicating that R91 and E106 in Orai1 are critical amino acids for pore formation, and they are equally important in both the SOCE reaction and thermal response. Furthermore, the thermal responses induced by STIM1/Orai1–R91W and STIM1/Orai1–E106A were even lower than those of the blank control STIM1/mCherry. The inhibitory effect of overexpressed Orai1 mutants on endogenous Orai1 ion channels may explain these results [40]. Additionally, E149 of Orai1, located in the loop2 region (Figure 7A), forms a salt bridge with the N–terminal R83 when Orai1 is activated and is crucial for transmitting conformational changes that gate the Orai1 ion channel [41]. Our study found that the thermal response and SOCE reaction of the single–gene mutant STIM1/Orai1–E149R was almost abolished, while there was no significant change in STIM1/Orai1–R83E. The double–gene mutant STIM1/Orai1–R83E–E149R partially restored the SOCE reaction and thermal response (Figure 7D–G). The various Orai1 mutants did not display any noticeable difference between the thermal response and SOCE reaction. This is different from STIM1 mutants, which exhibited both similarities and differences between thermal response and SOCE reaction.

### 3.7. Molecular Mechanism Analysis of the Different Thermal Response Patterns between STIM1/Orai1 and STIM1/Orai3

**Figure 8:**
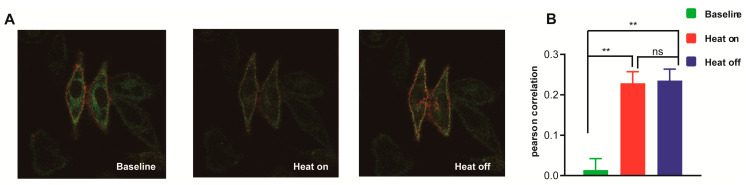
Co–localization analysis of STIM1 and Orai3 during thermal response. (**A**) Representative figures and (**B**) statistic analysis of the co–localization of STIM1 and Orai3 at the phase of baseline, heat–on and heat–off, respectively (*n* = 3 coverslips). The red color (mCherry) represents the Orai3 protein, while the green color (GFP) represents the STIM1 protein. Data were expressed as x ± s. ** p < 0.01.

In our previous studies, we discovered that STIM1/Orai1 mainly facilitated the thermal response during cooling as heat–off response, while STIM1/Orai3 played a significant role in thermal response during both heating (heat–on response) and cooling [20,21]. The coupling mechanism of STIM1/Orai1 has been extensively studied, which revealed that its coupling is impeded at higher temperatures, and its inhibitory effect is reduced during cooling, leading to a heat–off response [20]. In this research, we focused on elucidating the coupling mechanism of STIM1/Orai3. Using confocal analysis, we found that STIM1/Orai3 exhibited comparable co–localization levels during both heating and cooling processes (Figure 8), providing an additional understanding of its molecular mechanism of thermal response.

**Figure 9:**
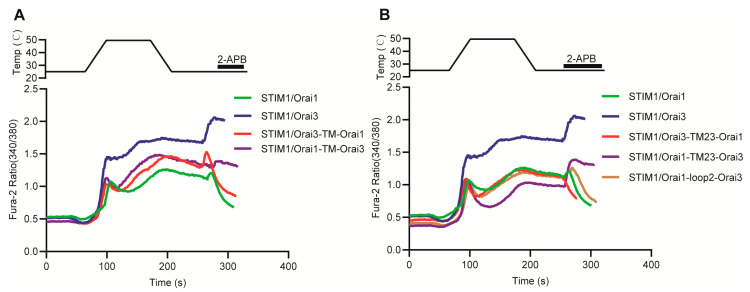
Distinct molecular mechanism analysis of thermal responses between STIM1/Orai1 and STIM1/Orai3. (**A**,**B**) Representative average traces of HEK293T cells transfected with indicated constructs in response to a heating pulse to around 50 °C and subsequent 2–APB (100 μM) in the presence of 1.3 mM Ca^2+^ using single–cell Ca^2+^ imaging. Each test was repeated 3 times.

To investigate the molecular mechanisms underlying the differential thermal responses mediated by STIM1/Orai1 and STIM1/Orai3, we generated chimeric mutation plasmids of Orai1 and Orai3 and performed thermo response analysis using 2–APB as an auxiliary detection tool (Figure 7A). Our results showed that STIM1/Orai1 primarily mediated heat–off response, which was characterized by a small peak of rapid activation followed by rapid inhibition upon exposure to 2–APB. In contrast, STIM1/Orai3 displayed both heat–on and heat–off response, and upon exposure to 2–APB, it exhibited further activation on the basis of heat–off response, leading to calcium ion influx (Figure 9). By substituting the TM region of Orai3 with that of Orai1 (referred to as Orai3–TM–Orai1) and co–transfecting it with STIM1, we observed only the heat–off response and a rapid inhibition pattern upon exposure to 2–APB, which closely resembles the response observed in STIM1/Orai1 transfected cells. This suggests that the main factor determining the thermal response characteristics of STIM1/Orai1 is the Orai1 TM region, and by deleting the Orai3 TM region, the unique heat–on response of STIM1/Orai3 can be eliminated. We attempted to replicate the thermal response characteristics of STIM1/Orai3 by replacing the TM region of Orai1 with that of Orai3 (Orai1–TM–Orai3) and co–transfecting it with STIM1. However, the results obtained were not consistent with our initial predictions, as we did not observe the anticipated heat–on response. Instead, only a heat–off response was detected, which resembled that of the STIM1/Orai1 response pattern. Interestingly, after thermal stimulation and subsequent exposure to 2–APB, Orai1–TM–Orai3 exhibited a further activation effect, similar to STIM1/Orai3. Based on the data obtained, it can be conjectured that the heat–on response may result from a combined effect of Orai3, as the heat–on response was eliminated in both Orai3–TM–Orai1 (lack TM domain of Orai3) and Orai1–TM–Orai3 (lack N and C terminals of Orai3) constructs.

To further narrow down the key region, we constructed a chimeric mutant named Orai3–TM23–Orai1. This mutant was designed to replace the TM23 region of Orai3 with the corresponding region from Orai1, which includes the second transmembrane, second loop, and third transmembrane regions. We found that the thermal response mediated by STIM1/Orai3–TM23–Orai1 was similar to that of STIM1/Orai1, primarily a heat–off response. It suggests that only TM23 elimination of Orai3 can also abolish the heat–on response, while the TM23 region of Orai1 is the key domain determining STIM1/Orai1–induced heat–off response. Nevertheless, when exposed to 2–APB after thermal stimulation, STIM1/Orai3–TM23–Orai1 was directly inhibited by it, without the small peak of rapid activation observed in STIM1/Orai1, suggesting another region of Orai1 is responsible for this 2–APB induced rapid activation after heat–off response. Similarly, replacing the TM23 region of Orai1 with that of Orai3, i.e., STIM1/Orai1–TM23–Orai3, did not significantly affect its thermal response but resulted in a response pattern to 2–APB similar to that of STIM1/Orai3. We further pinpointed the region by replacing loop2 in Orai1 with that from Orai3 (STIM1/Orai1–loop2–Orai3), which did not significantly differ from STIM1/Orai1 in terms of thermal response or the response to 2–APB after thermal stimulation. This contrasts with previous reports that substitution of loop2 could reverse the response pattern for 2–APB between Orai1 and Orai3 [42]. In summary, our study indicates that the distinct thermal response characteristics and response patterns to 2–APB observed in STIM1/Orai1 and STIM1/Orai3 are attributable to disparate molecular mechanisms. TM23 region of Orai1 was identified as the key domain determining the STIM1/Orai1 thermal response pattern, while the heat–on and heat–off response pattern of STIM1/Orai3 resulted from a combined effect of various domains of Orai3.

## 4. Discussion

Thermal sensation is a vital function of STIM1. In our present study, we extensively analyzed the temperature sensitivity mechanism of STIM1 from the N–terminal to the C–terminal and identified the key temperature–sensitive sites of STIM1. Furthermore, we utilized the STIM1–induced SOCE response as a control to delineate the similarities and differences between thermal response and SOCE reaction. STIM1 (209–685)/Orai1 elicited a thermal response but not SOCE, which provides compelling evidence that STIM1 activation by temperature does not require the N–terminal of STIM1 and is not reliant on calcium release from the ER, which needs EF–SAM domain. Furthermore, we found that STIM1 (209–685) is the shortest truncation capable of inducing a heat response. When we further truncated the TM region of STIM1 to create STIM1–CT (233–685), no thermal response was observed. This discovery highlights the crucial role of the TM region in STIM1’s thermal sensation, although there exists the possibility that deletion of the TM region may destroy the fundamental function of STIM1. Previous research has shown that the TM region of STIM1 underwent structural rearrangements upon adopting an activated conformation, which subsequently triggered further conformational alterations in the cytosolic juxtamembrane region. Specifically, this led to the release of the STIM Orai–activating region (SOAR) from its interaction with the coiled–coil 1 (CC1) regions, allowing for physical extension of the STIM1 cytoplasmic domain across the gap [43]. Lower calcium influx was found for STIM1 (209–685)/Orai1 when changing Ca^2+^ concentration compared to the cytosolic protein STIM1–CT (233–685)/Orai1 at room temperature. This suggests that apart from working in conjunction with the luminal region to maintain STIM1 in an inactive state at rest [44]. TM region can also exhibit an inhibitory effect in other mechanisms. While STIM1 (209–685)/Orai1 showed a significant increase in basal calcium influx at higher temperatures, which was opposite to STIM1–CT (233–685)/Orai1 (Figure 2E), indicating that the TM region has a promoting effect on the relief of STIM1 inhibition when the temperature is elevated. The function of the TM region also highlights the crucial role of anchoring to the ER membrane in STIM1–dependent temperature sensation. Researchers have reported that the N234 residue, which marks the emergence of the cytosolic portion of STIM1 from the ER membrane, exhibited significant resonance changes upon STIM1 adopting an activated conformation. This finding aligns with our own investigation, which determined that the STIM1–N234E mutation impairs the SOCE response (Figure 3A,B) [26]. The normal thermal response of STIM1–N234E/Orai1 lends support to the notion that STIM1 activation via heat is not primarily dictated by ER membrane cues as SOCE. Additionally, we observed that the STIM1–C227W mutation, located in the transmembrane region, triggers constitutive calcium influx when expressed alongside Orai1. However, our study showed that both the thermal response and SOCE were impaired in this context, likely due to STIM1–C227W/Orai1 already being maximally activated and thus unresponsive to further temperature–induced activation.

Elevated temperature can decrease the inhibitory effect of CC1–SOAR, resulting in higher basal levels and calcium influx of CC1–SOAR/Orai1 upon 0 mM Ca^2+^ to 1.3 mM Ca^2+^ at 37 °C compared to 25 °C. We reasoned that CC1–SOAR is a direct–acting site for temperature. This was further corroborated by the destroyed heat–off response in STIM1–R426L/Orai1 transfected cells, which mutation could strengthen the inhibitory effect coming from CC1–SOAR. Although CC1–SOAR could be directly activated by temperature, leading to STIM1 activation, STIM1/Orai1 primarily shows a heat–off response during cooling rather than heating (Figure 1). This is mainly due to the inhibitory effect of heat on the interaction between SOAR and Orai1 [20] (Figure 5B,D). The STIM1–SOAR2 chimera showed a lack of both SOCE and thermal sensitivity, indicating the specific involvement of SOAR1 in STIM1. The STIM1–G379E mutation located in sα1 of SOAR1 resulted in a loss of SOCE but retained thermal response, while STIM1–F394 mutations located in sα4 showed similar trends for both thermal response and SOCE. It has been reported that sα1 is responsible for interacting with Orai1 [31], while sα4 mainly contributes to activating the Orai1 channel [37]. Therefore, we hypothesized that temperature may enhance the interaction between STIM1 and Orai1, which is stronger than that in the SOCE response, leading to the retention of normal thermal response for the STIM1–G379E mutation. However, for mutations related to activating the Orai1 channel in STIM1, such as STIM1–F394H/L, the thermal response could not be restored. Additionally, STIM2 is a useful auxiliary means for determining whether the K domain of STIM1 plays a crucial role in directly sensing temperature. This is due to the fact that, despite having higher homology with STIM1, no thermal sensation was observed for STIM2. Previous studies showed that the K domain of STIM1 plays a crucial role in facilitating STIM1 activation, promoting its translocation to the PM and interacting with Orai1, whereas its role in STIM2 is less significant [45]. STIM1–K2 rescued part of the thermal response of STIM1–ΔK, which suggests that the K domain may only be responsible for mediating the targeting of STIM1 to the PM rather than having a direct temperature–sensing role. Because if the K domain of STIM1 is responsible for directly activating STIM1, STIM1–K2 would be thermal–insensitive. Adding the K2 domain to STIM1–ΔK may facilitate the targeting of STIM1 to the ER–PM junction, similar to the K domain of STIM1, and the K domain–mediated targeting of STIM1 to PM can potentially be enhanced with elevated temperatures.

STIM1 can be activated by either temperature or the release of Ca^2+^ from the ER, resulting in thermal response or store–operated Ca^2+^ entry (SOCE), respectively. Therefore, it is critical to identify similarities and differences of Orai1 for its interaction with STIM1 under these two types of stimuli. In our research, we selected representative Orai1 mutations and found comparable response trends for thermal sensation and SOCE. These findings suggest that activation modes of STIM1 may not significantly impact its interaction with the key sites of Orai1. However, The distinct characteristics of Orai1 and Orai3 resulted in different thermal response patterns between STIM1/Orai1 and STIM1/Orai3. High temperatures inhibited the STIM1–Orai1 interaction [20], while comparable co–localization of STIM1 and Orai3 was observed during both heat–on and heat–off responses (Figure 8). Previous studies have shown that loop2 inhibits the coupling of Orai1 with STIM1 [46], and STIM1 couples more efficiently in the presence of Orai3–loop2 than Orai1–loop2 [29]. But we did not see heat–on response even having loop2 of Orai3 in Orai1 (Orai1–loop2–Orai3, Orai1–TM23–Orai3 and Orai1–TM–Orai3), suggesting other mechanisms exist for the heat–on response of Orai3. Additionally, Zhang et al. replaced the middle two transmembrane segments of Orai1 (TM23) with that from Orai3 as Orai1–TM23–Orai3 chimera, was capable of generating 2–APB–evoked Ca^2+^ influx, comparable to Orai3. Narrowing down the replacement area to the Orai1–loop2–Orai3 chimera, a partial response to 2–APB was observed [42]. We found that 2–APB had an activating effect on Orai1–TM23–Orai3 when added after thermal stimulation. However, there was no discernible difference in 2–APB response between Orai1–loop2–Orai3 and Orai1, which contrasts with previous findings. All these results suggest that differences exist between 2–APB and temperature–mediated responses for STIM1/Orai1 and STIM1/Orai3.

By utilizing single–cell calcium imaging and confocal analysis, this study expands upon previous research to uncover the underlying mechanisms of STIM1 temperature sensation. Further experiments and alternative methods will still be required to conduct a more detailed investigation of this mechanism.

## 5. Conclusions

Our findings provide some important molecular mechanisms underlying STIM1–induced thermal response, as well as the interaction mechanisms between STIM1 and Orai1 or Orai3 following activation by temperature.

## Data Availability

Data are available upon request.

## References

[1] Dhaka A., Viswanath V., Patapoutian A. (2006). Trp ion channels and temperature sensation. Annu. Rev. Neurosci..

[2] Caterina M.J., Schumacher M.A., Tominaga M., Rosen T.A., Levine J.D., Julius D. (1997). The capsaicin receptor: A heat–activated ion channel in the pain pathway. Nature.

[3] Caterina M.J., Leffler A., Malmberg A.B., Martin W.J., Trafton J., Petersen–Zeitz K.R., Koltzenburg M., Basbaum A.I., Julius D. (2000). Impaired nociception and pain sensation in mice lacking the capsaicin receptor. Science.

[4] Caterina M.J., Rosen T.A., Tominaga M., Brake A.J., Julius D. (1999). A capsaicin–receptor homologue with a high threshold for noxious heat. Nature.

[5] Vriens J., Owsianik G., Hofmann T., Philipp S.E., Stab J., Chen X., Benoit M., Xue F., Janssens A., Kerselaers S. (2011). TRPM3 is a nociceptor channel involved in the detection of noxious heat. Neuron.

[6] Peier A.M., Reeve A.J., Andersson D.A., Moqrich A., Earley T.J., Hergarden A.C., Story G.M., Colley S., Hogenesch J.B., McIntyre P. (2002). A heat–sensitive TRP channel expressed in keratinocytes. Science.

[7] Watanabe H., Vriens J., Suh S.H., Benham C.D., Droogmans G., Nilius B. (2002). Heat–evoked activation of TRPV4 channels in a HEK293 cell expression system and in native mouse aorta endothelial cells. J. Biol. Chem..

[8] Tan C.H., McNaughton P.A. (2016). The TRPM2 ion channel is required for sensitivity to warmth. Nature.

[9] McKemy D.D., Neuhausser W.M., JuLius D. (2002). Identification of a cold receptor reveals a general role for TRP channels in thermosensation. Nature.

[10] Story G.M., Peier A.M., Reeve A.J., Eid S.R., Mosbacher J., Hricik T.R., Earley T.J., Hergarden A.C., Andersson D.A., Hwang S.W. (2003). ANKTM1, a TRP–like Channel Expressed in Nociceptive Neurons, Is Activated by Cold Temperatures. Cell.

[11] Kashio M., Tominaga M. (2022). TRP channels in thermosensation. Curr. Opin. Neurobiol..

[12] Zhang H., Wang C., Zhang K., Kamau P.M., Luo A., Tian L., Lai R. (2022). The role of TRPA1 channels in thermosensation. Cell Insight.

[13] Vandewauw I., De Clercq K., Mulier M., Held K., Pinto S., Van Ranst N., Segal A., Voet T., Vennekens R., Zimmermann K. (2018). A TRP channel trio mediates acute noxious heat sensing. Nature.

[14] Meents J.E., Ciotu C.I., Fischer M.J.M. (2019). TRPA1: A molecular view. J. Neurophysiol..

[15] Moqrich A., Hwang S.W., Earley T.J., Petrus M.J., Murray A.N., Spencer K.S., Andahazy M., Story G.M., Patapoutian A. (2005). Impaired thermosensation in mice lacking TRPV3, a heat and camphor sensor in the skin. Science.

[16] Huang S.M., Li X., Yu Y., Wang J., Caterina M.J. (2011). TRPV3 and TRPV4 ion channels are not major contributors to mouse heat sensation. Mol. Pain.

[17] Lee H., Iida T., Mizuno A., Suzuki M., Caterina M.J. (2005). Altered thermal selection behavior in mice lacking transient receptor potential vanilloid 4. J. Neurosci..

[18] Yarmolinsky D.A., Peng Y., Pogorzala L.A., Rutlin M., Hoon M.A., Zuker C.S. (2016). Coding and plasticity in the mammalian thermosensory system. Neuron.

[19] Paricio–Montesinos R., Schwaller F., Udhayachandran A., Rau F., Walcher J., Evangelista R., Vriens J., Voets T., Poulet J.F.A., Lewin G.R. (2020). The Sensory Coding of Warm Perception. Neuron.

[20] Xiao B., Coste B., Mathur J., Patapoutian A. (2011). Temperature–dependent STIM1 activation induces Ca^2+^ influx and modulates gene expression. Nat. Chem. Biol..

[21] Liu X., Wang H., Jiang Y., Zheng Q., Petrus M., Zhang M., Zheng S., Schmedt C., Dong X., Xiao B. (2019). STIM1 thermosensitivity defines the optimal preference temperature for warm sensation in mice. Cell Res..

[22] Roos J., DiGregorio P.J., Yeromin A.V., Ohlsen K., Lioudyno M., Zhang S., Safrina O., Kozak J.A., Wagner S.L., Cahalan M.D. (2005). STIM1, an essential and conserved component of store–operated Ca^2+^ channel function. J. Cell Biol..

[23] Vig M., Peinelt C., Beck A., Koomoa D.L., Rabah D., Koblan–Huberson M., Kraft S., Turner H., Fleig A., Penner R. (2006). CRACM1 Is a Plasma Membrane Protein Essential for Store–Operated Ca^2+^ Entry. Science.

[24] Rubaiy H.N. (2023). ORAI Calcium Channels: Regulation, Function, Pharmacology, and Therapeutic Targets. Pharmaceuticals.

[25] Novello M.J., Zhu J., Feng Q., Ikura M., Stathopulos P.B. (2018). Structural elements of stromal interaction molecule function. Cell Calcium.

[26] Ma G., Wei M., He L., Liu C., Wu B., Zhang S.L., Jing J., Liang X., Senes A., Tan P. (2015). Inside–out Ca^2+^ signalling prompted by STIM1 conformational switch. Nat. Commun..

[27] Wang Y., Deng X., Zhou Y., Hendron E., Mancarella S., Ritchie M.F., Tang X.D., Baba Y., Kurosaki T., Mori Y. (2009). STIM Protein Coupling in the Activation of Orai Channels. Proc. Natl. Acad. Sci. USA.

[28] Korzeniowski M.K., Manjarres I.M., Varnai P., Balla T. (2010). Activation of STIM1–Orai1 involves an intramolecular switching mechanism. Sci. Signal..

[29] Park C.Y., Hoover P.J., Mullins F.M., Bachhawat P., Covington E.D., Raunser S., Walz T., Garcia K.C., Dolmetsch R.E., Lewis R.S. (2009). STIM1 clusters and activates CRAC channels via direct binding of a cytosolic domain to Orai1. Cell.

[30] Muik M., Fahrner M., Schindl R., Stathopulos P., Frischauf I., Derler I., Plenk P., Lackner B., Groschner K., Ikura M. (2011). STIM1 couples to ORAI1 via an intramolecular transition into an extended conformation. EMBO J..

[31] Zheng S., Ma G., He L., Zhang T., Li J., Yuan X., Nguyen N.T., Huang Y., Zhang X., Gao P. (2018). Identification of molecular determinants that govern distinct STIM2 activation dynamics. PLoS Biol..

[32] Shrestha N., Hye–Ryong Shim A., Maneshi M.M., See–Wai Yeung P., Yamashita M., Prakriya M. (2022). Mapping interactions between the CRAC activation domain and CC1 regulating the activity of the ER Ca^2+^ sensor STIM1. J. Biol. Chem..

[33] Rathner P., Fahrner M., Cerofolini L., Grabmayr H., Horvath F., Krobath H., Gupta A., Ravera E., Fragai M., Bechmann M. (2021). Interhelical interactions within the STIM1 CC1 domain modulate CRAC channel activation. Nat. Chem. Biol..

[34] Fahrner M., Muik M., Schindl R., Butorac C., Stathopulos P., Zheng L., Jardin I., Ikura M., Romanin C. (2014). A Coiled–coil Clamp Controls Both Conformation and Clustering of Stromal Interaction Molecule 1 (STIM1). J. Biol. Chem..

[35] Butorac C., Muik M., Derler I., Stadlbauer M., Lunz V., Krizova A., Lindinger S., Schober R., Frischauf I., Bhardwaj R. (2019). A novel STIM1–Orai1 gating interface essential for CRAC channel activation. Cell Calcium.

[36] Korzeniowski M.K., Baird B., Holowka D. (2016). STIM1 activation is regulated by a 14 amino acid sequence adjacent to the CRAC activation domain. AIMS Biophys..

[37] Wang X., Wang Y., Zhou Y., Hendron E., Mancarella S., Andrake M.D., Rothberg B.S., Soboloff J., Gill D.L. (2014). Distinct Orai–coupling domains in STIM1 and STIM2 define the Orai–activating site. Nat. Commun..

[38] Yamashita M., Ing C.E., Yeung P.S., Maneshi M.M., Pomes R., Prakriya M. (2020). The basic residues in the Orai1 channel inner pore promote opening of the outer hydrophobic gate. J. Gen. Physiol..

[39] Cai X., Zhou Y., Nwokonko R.M., Loktionova N.A., Wang X., Xin P., Trebak M., Wang Y., Gill D.L. (2016). The Orai1 Store–operated Calcium Channel Functions as a Hexamer. J. Biol. Chem..

[40] Deng X., Wang Y., Zhou Y., Soboloff J., Gill D.L. (2009). STIM and Orai: Dynamic intermembrane coupling to control cellular calcium signals. J. Biol. Chem..

[41] Dong H., Zhang Y., Song R., Xu J., Yuan Y., Liu J., Li J., Zheng S., Liu T., Lu B. (2019). Toward a Model for Activation of Orai Channel. iScience.

[42] Zhang S.L., Kozak J.A., Jiang W., Yeromin A.V., Chen J., Yu Y., Penna A., Shen W., Chi V., Cahalan M.D. (2008). Store–dependent and –independent modes regulating Ca^2+^ release–activated Ca^2+^ channel activity of human Orai1 and Orai3. J. Biol. Chem..

[43] Zhou Y., Srinivasan P., Razavi S., Seymour S., Meraner P., Gudlur A., Stathopulos P.B., Ikura M., Rao A., Hogan P.G. (2013). Initial activation of STIM1, the regulator of store–operated calcium entry. Nat. Struct. Mol. Biol..

[44] Ma G., Zheng S., Ke Y., Zhou L., He L., Huang Y., Wang Y., Zhou Y. (2017). Molecular Determinants for STIM1 Activation During Store– Operated Ca^2+^ Entry. Curr. Mol. Med..

[45] Bhardwaj R., Muller H.M., Nickel W., Seedorf M. (2013). Oligomerization and Ca^2+^/calmodulin control binding of the ER Ca2+–sensors STIM1 and STIM2 to plasma membrane lipids. Biosci. Rep..

[46] Fahrner M., Pandey S.K., Muik M., Traxler L., Butorac C., Stadlbauer M., Zayats V., Krizova A., Plenk P., Frischauf I. (2018). Communication between N terminus and loop2 tunes Orai activation. J. Biol. Chem..

